# The Influence of Closed-Cell W-Shaped Liner Parameters on the Penetration Performance of Integral Annular Shaped Charge

**DOI:** 10.3390/ma15207155

**Published:** 2022-10-14

**Authors:** Zhilin Yang, Jianping Fu, Fudi Liang, Likui Yin, Kai Ren, Hao Yuan, Hongxin Li, Taiyong Zhao, Zhigang Chen

**Affiliations:** 1 College of Mechatronic Engineering, North University of China, Taiyuan 030051, China; 2 National Defense Key Laboratory of Underground Damage Technology, North University of China, Taiyuan 030051, China; 3 Shanxi Jiangyang Chemical Co., Ltd., Taiyuan 030051, China; 4 Institute of Chemical Defense, Academy of Military Sciences, Beijing 102205, China

**Keywords:** W-shaped charge liner, annular shaped charge, numerical simulation

## Abstract

To further enhance the hole-opening ability of the metal jet formed by the annular shaped charge on the armored steel target, a new annular shaped charge structure of a closed-cell W-shaped charge liner is designed based on a certain penetration depth. The impact of the length-diameter ratio of the charge, the inverted cone angle, and the cone angle of the liner on the opening diameter of the annular metal jet are studied through the orthogonal optimization of the annular shaped charge structures carried out by numerical simulation, which shows that the inverted cone angle and the cone angle of the liner are among the main factors that affect penetration depth and the opening diameter of the annular jet, respectively. According to this, an optimum annular charge structure considering both penetration depth and opening diameter is designed and tested by experiments. According to the results, the optimized annular jet records an opening diameter of 0.83 CD (Charge Diameter) when it penetrates the armored steel target with a thickness of 0.53 CD. The experimental results are consistent with the numerical simulation. The research results have certain practical engineering significance for guiding the design of the front-stage annular shaped charge structure of the multi-effect destructive warhead.

## 1. Introduction

In general, the front stage of the tandem assault charge is a shaped charge, and the rear stage is a follow-on bullet. However, the jet hole diameter formed by the traditional shaped charge is small, which cannot meet the requirements of the follow-on large caliber rear stage bullet. It is urgent to carry out research on large hole opening technology. The annular jet hole diameter formed by the annular shaped charge is large, which can achieve circular cutting and has attracted a lot of research from scholars around the world: Leidel D. J. et al. designed an annular shaped charge structure which obtained the annular jet by adding a steel cylinder to the charge [1]. Chick M. C. et al. improved the structure of the annular shaped charge, enhancing the uniformity of the overall velocity and the flight stability of the annular jet [2]. Meister J. et al. conducted experimental research on two annular shaped charge structures with one overturning along the center and another along the outside [3]. Konig P. J. et al. designed an annular liner structure that can form annular EFP that overturns the liner based on the unequal wall thickness at different positions of the liner [4]. Richard F et al. introduced several annular cutters with steel reinforced concrete and brick walls as primary targets designed by the U.S. ERDC (Army Equipment Research and Development Center) on the 25th ICB (International Conference on Ballistics) [5]. Towards the same target, LI Y L et al. designed an annular shaped charge structure that formed a two-layer annular EFP [6]. Wang C et al. proposed the W-typed shaped charge and put forward the principle of designing the inner and outer liners with equal impulse, on which numerical simulation and experimental research were carried out [7]. Fu L. et al. brought forward a new charge annular cutting structure with research on the processing of this structure and conducted numerical simulations of the jet shaping and penetration into the two-layer target by LS-DYNA [8]. With numerical simulation and experimental research, Lu Y et al. advanced the annular cutter and determined the cutting angle that can achieve the machine’s ideal cutting effect [9]. Zhang Z et al. set up the SPH (Smoothed Particle Hydrodynamics) model of the annular jet penetrating the steel target and then made comparisons between the numerical results and experimental data [10]. Xu W et al. optimized the parameters of the liner in the annular shaped charge structure by numerical simulation and experimental verification, with the optimal value of the parameters being obtained [11,12,13]. To get a better destructive effect of the metal jet on the double-hull submarine, Hu Z et al. designed a new combined annular shaped charge structure that can form rod-liked jets and annular jet penetrators [14]. Si Y R et al. devised a combined EFP (explosively formed penetrator) that can produce an annular jet that causes a large opening and center to the concrete walls [15].

Despite the distinct structures designed by researchers to study the properties of the annular jet, most of the structures under current research have been disadvantaged by the shallow penetration depth or the small opening diameters when they form the annular jet to penetrate armored steel targets. This article designs a new annular shaped charge structure for a closed-cell W-shaped liner and conducts the orthogonal optimization of the annular shaped charge structures carried out by the numerical simulation to study the influence law of the length-diameter ratio of the charge, the inverted cone angle, and the cone angle of the liner on the penetration depth and opening diameter of the annular metal jet. In the end, an optimum annular shaped charge structure is obtained with good performance in penetration depth and opening diameter after optimization, which has been verified by experiments.

The research results are of great value in guiding the structural design of the front-stage annular shaped charge structure of the multi-effect destructive warhead.

## 2. Simulation Calculation

### 2.1. Simulation Model

**Figure 1:**
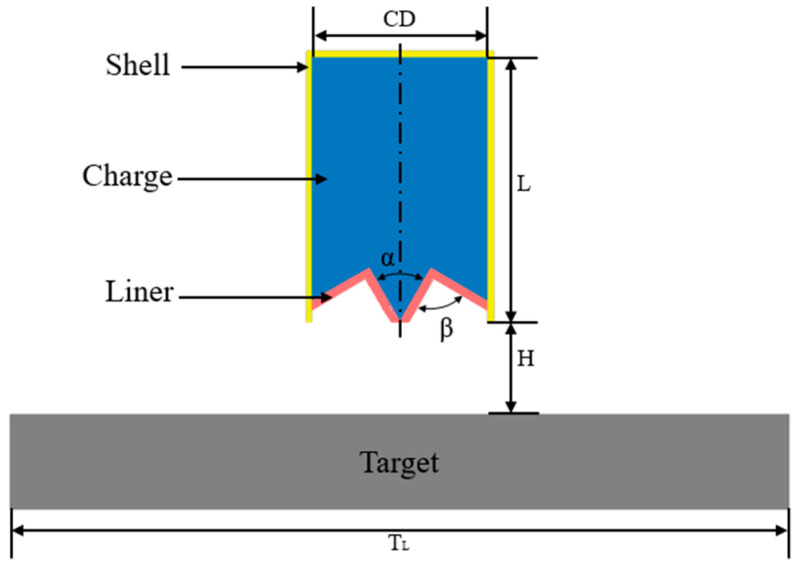
Schematic of the structural simulation model of an annular shaped charge.

A two-dimensional model of the annular shaped charge structure is set up by the LS-DYNA, consisting of the liner, the charge, and the shell as well as the target, as shown in Figure 1. The charge diameter-CD is 114 mm; the shell thickness is 3 mm; and the charge height at the top of the liner is 34.66 mm. The liner has a uniform wall thickness of 5 mm. L is the charge length, α is the inverted cone angle, and β is the cone angle of the liner. The target plate is cylindrical-shaped and its distance H to the mouth of the shaped charge is 60 mm (0.53 CD). The Euler algorithm is used to calculate the liner and the charge with a grid size of 1 × 1 mm; the Lagrange algorithm is used for the shell and target plate with a mesh size of 0.5 × 0.5 mm; the fluid-structure coupling is used between them with cm-g-µs as the system of the unit. In the case of any movement of the target plate during calculation that affects an accurate calculation of the data simulation, a fixed constraint, boundary-spc-set, is applied to the symmetrical plane of the target plate.

### 2.2. Material Parameters

The charge, the liner, the shell, and the target are made of 8701 explosives, red copper, steel, and armored steel, with the material parameters shown in Table 1 and Table 2.

The description of 8701 explosive is carried out jointly by the high-explosive-burn model and the JWL state equation, the specific parameters of which can be found in Table 1. The JWL state equation can better describe the movement process of detonation products after initiating the explosives and is widely used in the numerical simulation of explosive detonation and explosion drive. It is expressed as follows:

(1) $$P=A\left(1-\frac{\omega }{R_{1}V}\right)e^{-R_{1}V}+B\left(1-\frac{\omega }{R_{2}V}\right)e^{-R_{2}V}+\frac{\omega E}{V}$$

In this equation, *P* is the isentropic pressure; *V* is the relative volume of detonation products; *A*, *B*, *R*_1_, *R*_2_, and *ω* are the inputted parameters; and *E* is the internal volume energy.

The red copper, steel, and armored steel are represented by the Johnson–Cook constitutive model with specific parameters shown in Table 2. The Johnson–Cook model can show how the load pressure, temperature, plastic strain, strain rate, and material strength influence each other, which can be expressed as follows:

(2) $$\sigma_{e}=\left(A+B\varepsilon_{p}^{n}\right)\left(1+CIn\frac{\varepsilon }{\varepsilon_{0}}\right)\left(1-T^{*m}\right)$$

In this equation, *A* is the static yield stress; *B* is the hardening constant; *C* is the strain rate constant; *n* is the hardening exponent; *m* is the thermal softening exponent; $\sigma_{e}$ is the dynamic yield stress; $\varepsilon_{p}$ is the effective plastic strain; ε and $\varepsilon_{0}$ are the current strain rate; $T^{*m}=\left(T-T_{r}\right)\left(T_{m}-T_{r}\right)$ is the dimensionless temperature; T is the current temperature; $T_{r}$ is the reference temperature; and $T_{m}$ is the melting temperature of the material.

## 3. Scheme Design of Orthogonal Optimization Simulation and Result Analysis

### 3.1. Orthogonal Optimization Scheme Design

With the same charge height of the liner, orthogonal optimization is employed to select the best match among the 4 × 4 influencing factors. The penetration depth (P) and the opening diameter, D_h_, are two indexes to evaluate the optimization design, and the L_16_(4^4^) orthogonal table is used in the simulation. Because the annular jet formed when α > 80°, β < 80°, and β > 110° is not straight, it has poor penetration ability, and the charge energy is poorly utilized when L/CD > 2.0. Hence, there is no design under these conditions. The values of the various factor levels are shown in Table 3 [12].

### 3.2. Simulation Results and Analysis

A numerical simulation of the ultimate penetration depth is carried out for the armored steel target with Ø 150 × 80 mm when H = 0.53 CD. The calculation results of the orthogonal optimization simulation are shown in Table 4. The K1, K2, K3, K4, and range S are calculated at each level. The time node of 250 μs is selected to analyze the target plate (the annular jet velocity of all schemes is 0). The data processing and calculation results are shown in Table 5. Ki is the sum of various indicators corresponding to each factor at orthogonal level i.

From the range S in Table 5 the following can be concluded:

(1) The factors affecting penetration depth can be ranked in descending order of their influence: the inverted cone angle α, the cone angle of liner β, and the charge length-diameter ratio L/CD.
(2) The factors affecting opening diameters can be ranked in descending order of their influence: the cone angle of the liner β, the inverted cone angle α, and the charge length-diameter ratio L/CD.

**Figure 2:**
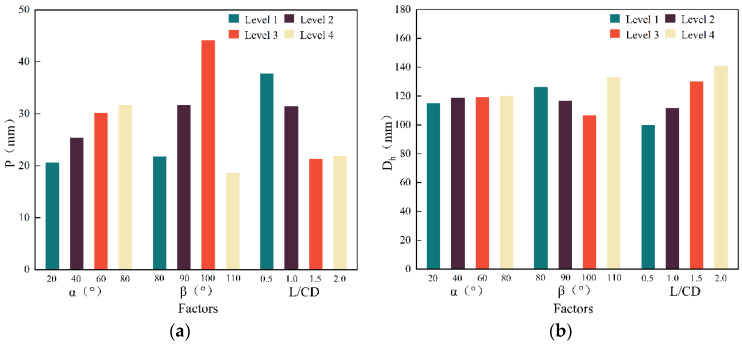
Variation curve of various indexes of annular jet penetrating the target plate with various factors: (**a**) The change in penetration depth with different factor levels; (**b**) The change in opening diameters with different factor levels.

For a more comprehensive analysis, the changes in each index with the level of factors are graphically shown in Figure 2.

Conclusions can be drawn from Figure 2 and the simulation results as follows:

(1) The inverted cone angle is the main factor that influences the penetration depth. As the inverted cone angle increases, the penetration depth grows first and then goes down, reaching its maximum value when the inverted cone angle α = 60°. Therefore, an inverted cone angle α = 60° is selected in the new annular shaped charge structure;
(2) The cone angle of the liner is a major contributor to the opening diameter. A larger cone angle of the liner would result in a bigger opening diameter at a lower penetration depth. When it reaches about 100°, there is no big difference in the penetration depth. It would be a more ideal situation if the opening diameter was as large as possible. Therefore, the cone angle of the liner is taken as 90° in the new structure;
(3) The charge length-diameter ratio has less influence on the penetration depth and opening diameter than the previous two factors. Increasing the length-diameter ratio can speed up the jet and deepen the penetration depth. The increasing penetration depth would be slowed down in which few benefits would be found when the ratio is above 1.5 and the opening diameter is less affected by the charge length-diameter ratio compared with the other two. With all-rounded consideration, the length-diameter ratio of 1.5 is taken in the new structure;
(4) Given all of that, the optimum scheme parameters of the overall annular shaped charge structure of the new closed-cell W-shaped liner are inverted cone angle α = 60°, the cone angle of the liner β = 90°, and the charge length-diameter ratio L/CD = 1.5.

## 4. Optimization Scheme Simulation and Result Analysis

Numerical simulation and analysis are carried out towards the formation process of the annular jet with the optimum scheme and the penetration process of the armored steel target plate by the annular shaped charge structure.

### 4.1. Analysis of the Formation Process of the Annular Jet

**Figure 3:**
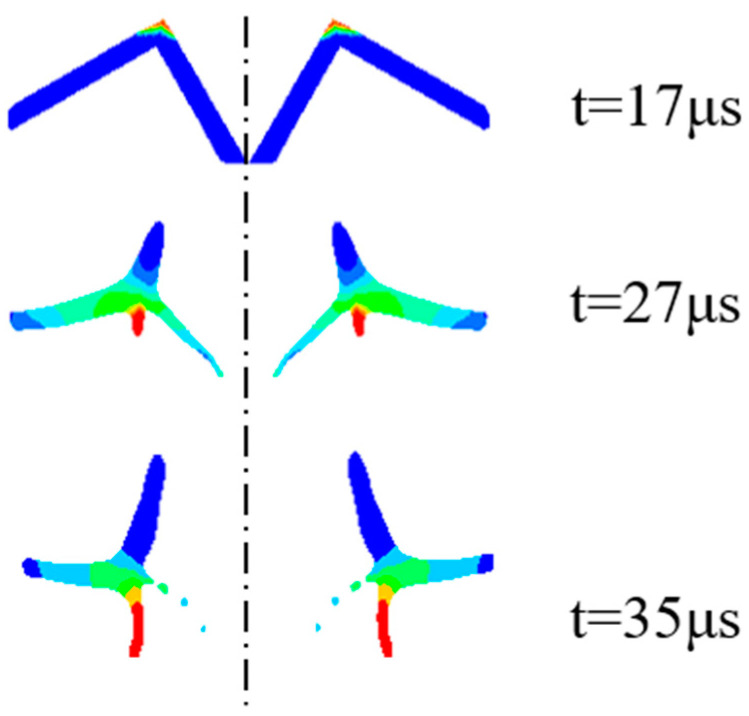
Formation process of the annular jet.

**Figure 4:**
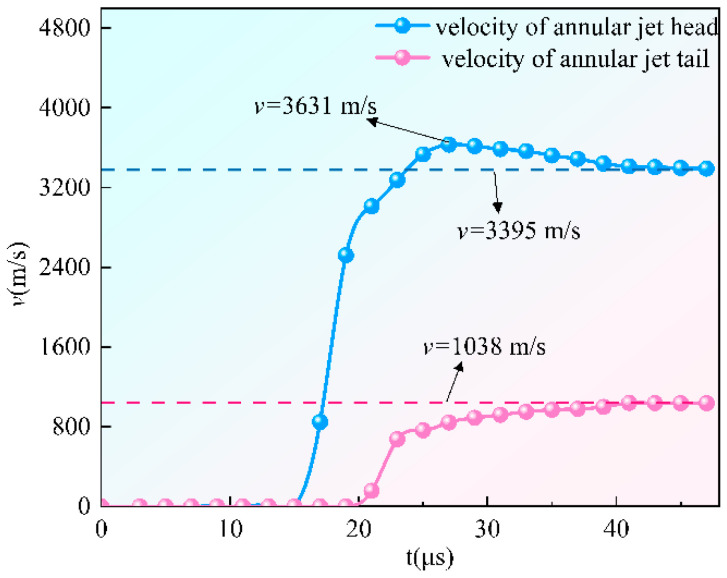
The velocity change curve of the jet hitting the head and tail during the annular jet formation process.

The annular jet formation process is shown in Figure 3, and the velocity change curve of the jet hitting the head and tail during the annular jet formation process can be found in Figure 4. It is initiated at the center point. When t = 17 μs, the detonation wave reaches the top of the liner, in which the W-shaped liner is crushed under the strong impact load, and the wave converges along the axis to form an annular jet. When t = 27 μs, the jet head grows rapidly, and the annular jet enters the stretching stage. When t = 35 μs, the jet is basically formed.

When t = 27 μs, the velocity of the jet head reaches a maximum of 3631 m/s and then slightly slows down, influenced by the detonation products, detonation pressure, and velocity gradient. It is stable at a basic speed of 3395 m/s when t = 41 μs. For the velocity of the jet tail, it starts to increase slowly when t = 21 μs and is stabilized at 1038 m/s when t = 41 μs.

**Figure 5:**
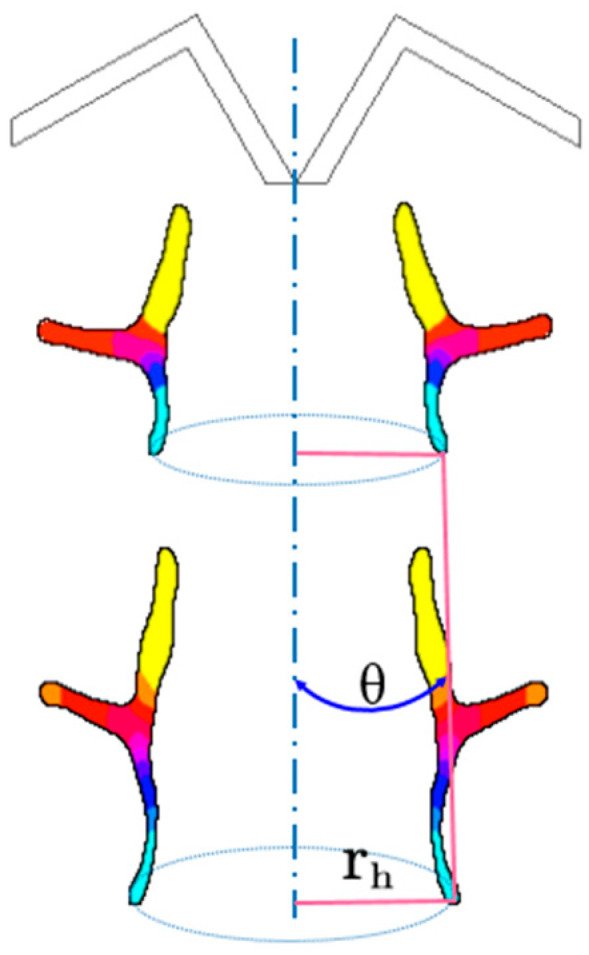
The relevant parameters of the W-shaped liner and the annular jet.

The relevant parameters of the W-shaped liner and the annular jet are shown in Figure 5, in which θ refers to the movement direction angle of the jet head (the angle between the connecting line of the jet head at the next tick of the clock and the jet head at the previous tick with the central axis), and r_h_ is the annular radius of the jet head.

**Figure 6:**
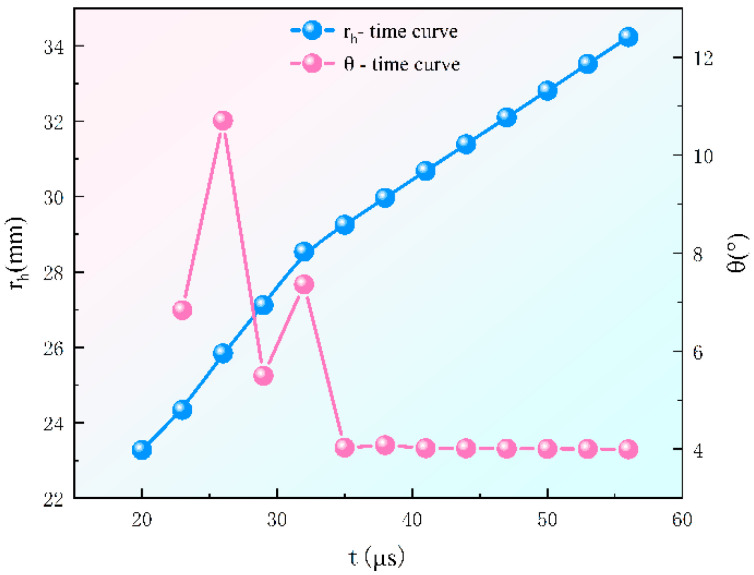
The curve of rh and θ over time.

The curve changes of r_h_ and θ over time are shown in Figure 6. From Figure 6, we found that when t = 20~35 μs, θ shows dynamic changes during which the jet head moves unstably, and the annular jet is not fully formed. When t > 35 μs, θ tends to be stable and the jet head moves in a straight line in the direction of 4° away from the axis, at which stage the annular jet is formed basically and r_h_ rises linearly. However, when t = 45 μs, the jet head is 61.37 mm away from the mouth of the liner, during which time the jet head is broken, and its penetration ability is cut down. As a result, in the new structure, the H of 60.0 mm (equal to 0.53 CD) is used.

### 4.2. Analysis of the Penetration Process

**Figure 7:**
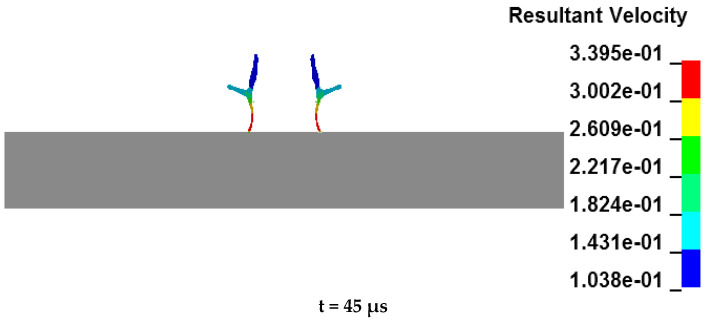

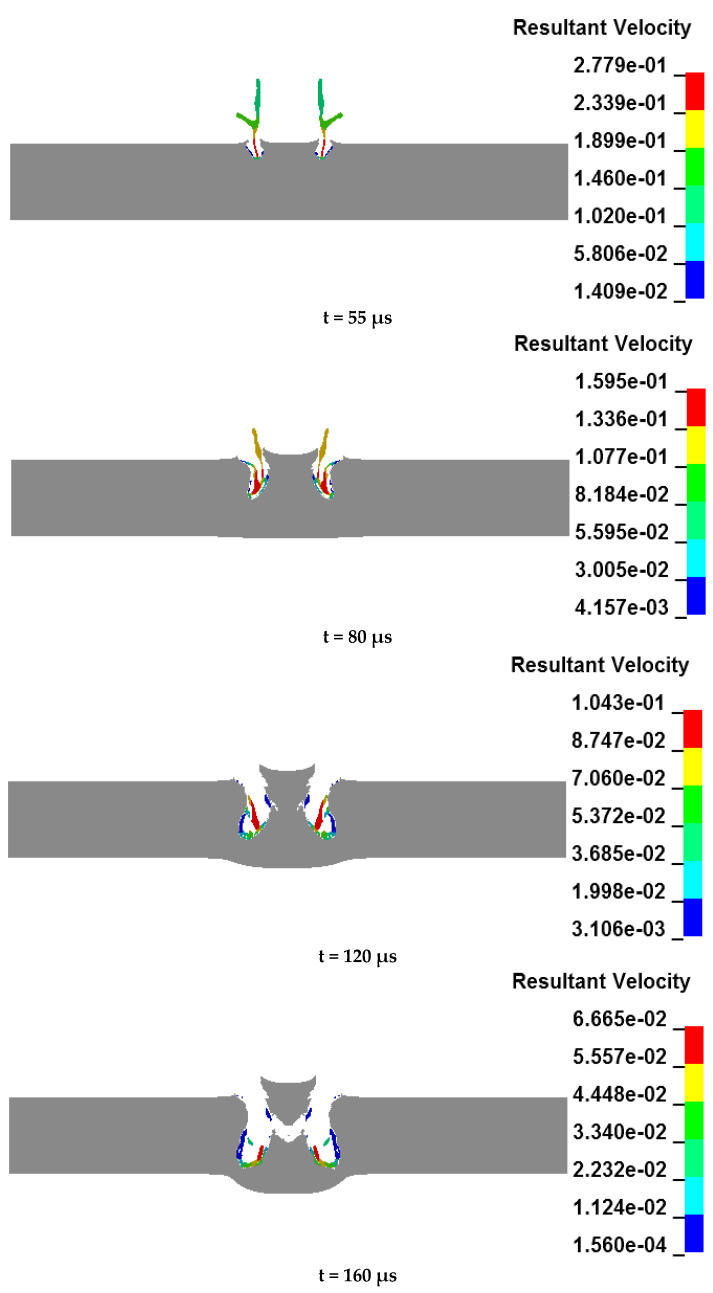

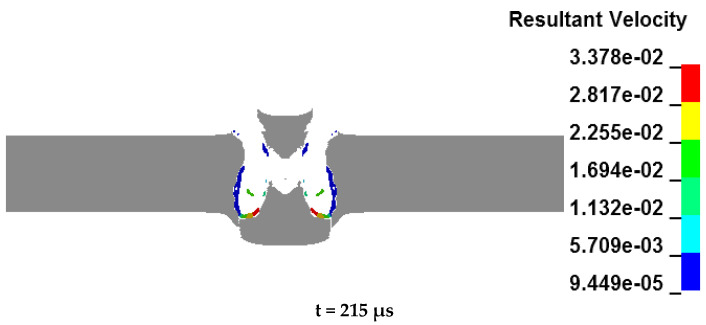
The penetration process of the annular jet to the target plate.

The process of the annular jet penetrating the target plate can be found in Figure 7. As can be seen from Figure 7, when t = 45 μs, the jet head contacts the target plate and the jet begins to penetrate the target plate, which is the opening stage of the jet [18]. When t reaches 45~80 μs, the maximum velocity of the jet head falls from 3395 m/s to 1595 m/s and the jet head completes the penetration process that goes about 48% of the total penetration depth, which is the stage of the jet head penetration; when t arrives at 80~160 μs, the jet head is completely abraded, and the penetration process is completed by the jet tail. The jet tail is slower than the jet head and is larger in terms of total mass but smaller than the head in overall energy. Meanwhile, the penetration depth of the jet tail is smaller, accounting for about 37% of the total penetration depth. It is the stage of the tail penetration. When t = 45~160 μs, it comes to the expansion stage of the jet penetration when the penetration goes deeper but the penetration aperture shows little change. When t = 160~215 μs, the back of the target plate bulges and deforms impacted by the jet, and a large shear force is generated in the target plate material below the penetration hole, which is too strong for the remaining thickness of the target plate to resist. Then the target plate shears and destroys at this position, which forms an annular plugging block that accounts for about 15% of the total penetration depth. It is called the plug stage of jet penetration.

**Figure 8:**
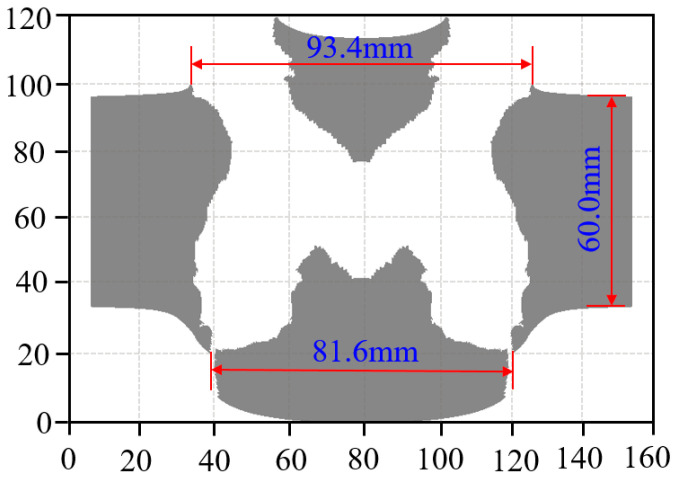
An effect drawing of the annular jet penetrating the target plate.

The optimal overall annular shaped charge structure can penetrate the armored steel target with a thickness of 60.0 mm, with the maximum remaining velocity of the annular jet recording 337.8 m/s after penetrating through the plate. The final penetration effect is shown in Figure 8, from which it can be seen that the opening aperture is 93.4 mm (equivalent to 0.83 CD) and the outlet aperture is 81.6 mm (equivalent to 0.72 CD). 

### 4.3. Experiment Verification

**Figure 9:**
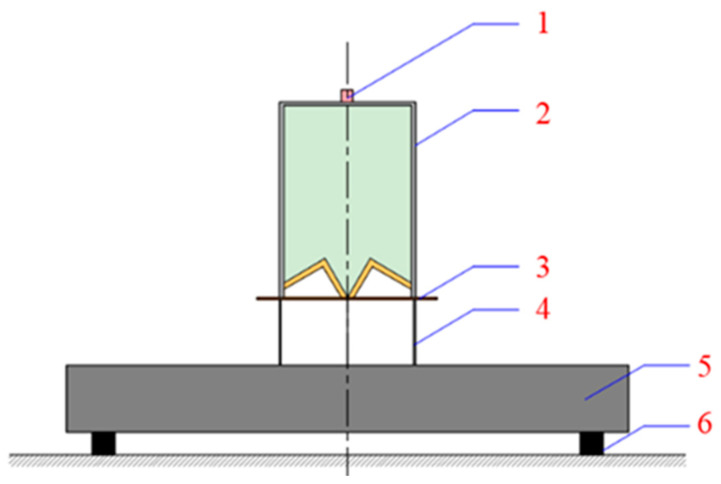
The schematic diagram of the test layout: **1**—detonator; **2**—annular shaped charge warhead; **3**—cardboard; **4**—deep fried drum; **5**—armored steel target plate; **6**—Support.

**Figure 10:**
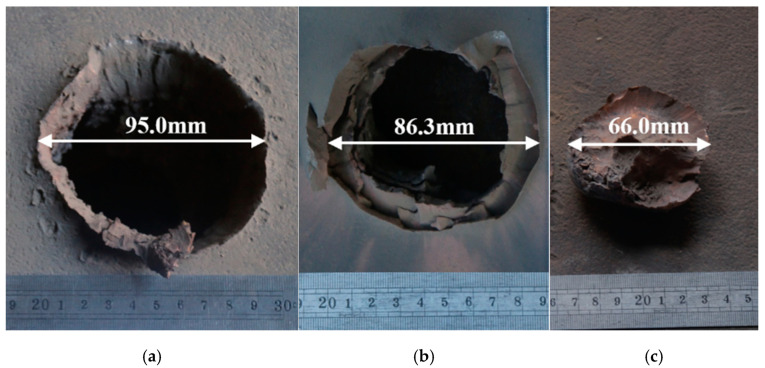
Diagram of test result: (**a**) the front of the target penetration entrance; (**b**) rear target penetration exit; and (**c**) plug block cut from the armored steel.

The schematic diagram of the test layout can be found in Figure 9. The structure after the above optimization design is taken as the test bomb structure, and the armor steel with a thickness of 60.0 mm is chosen as the target plate. The test explosion height of 60.0 mm is consistent with the simulation, in which the thickness of cardboard is 2 mm, and the height of the bursting height cylinder is 58 mm. Figure 10 shows the photos from the test of the plug blocks cut from the penetration inlet and outlet as well as the target plate taken after the static armor-piercing test. The test methods and procedures are as follows:

(1) Arrange the site according to the schematic diagram of the test layout;
(2) Test the detonating line to ensure that the line is unblocked;
(3) Place the test bomb and connect the detonating line;
(4) Detonates the test bomb and measure the hole diameter with a rigid ruler.

There is a certain thickness of red copper residue on the surface of the plug block cut from the armored steel target plate, which indicates that the annular jet has a certain accumulation effect when it penetrates the target plate end. Different from the jet formed by the single cone liner, the annular jet formed by the W-shaped liner has a large amount of energy released by the accumulation layer, which can produce a large shear force on the remaining target plate, causing a plug block forming on the target plate that is approximately circular in the upper surface with a diameter of 66.0 mm. Under the action of the annular jet, the penetration entrance of the target is extruded outward to form flanging, while the penetration exit of the target is deformed along the annular bulge by the shear action, and some parts are torn, which is in good agreement with the simulation results.

According to the comparison between the numerical simulation results and the test results in Table 6, the armored steel target with a thickness of 60.0 mm can be penetrated in both the numerical simulation and the test, and there is a deviation of 0.74% between the numerical simulation results of the opening diameter and the test results and a deviation of 5.45% between the numerical simulation results of the outlet aperture and the test results. The basic consistency between the numerical simulation results and the experimental results, by comparison, proves that the calculation method, material model, and related parameters used in the study are reasonable.

## 5. Conclusions

Through numerical simulation and experimental verification, this paper studies how the parameters of the closed-cell W-shaped liner influence the penetration performance of the integral annular shaped charge. The following conclusions can be drawn from the results as follows:

(1) The simulation analysis shows that the influence of various factors on the penetration depth is sequenced as: inverted cone angle > cone angle of the liner > charge length-diameter ratio, and that on the penetration depth can be ordered as: cone angle of the liner > inverted cone angle > charge length-diameter ratio. The inverted cone angle and the cone angle of the liner are the main influencing factors of penetration depth and opening aperture, respectively. The penetration depth first increases and then drops as the inverted cone angle becomes larger. The opening diameter becomes larger as the cone angle of the liner rises;
(2) The optimum overall annular shaped charge structure with both excellent penetration depth and an opening aperture is obtained through orthogonal optimization design, in which the inverted cone angle is 60°, the cone angle of the cover is 90°, and the length to diameter ratio of the charge is 1.5;
(3) The static armor-piercing test results of the optimized annular shaped charge structure show that the opening diameter can reach 95.0 mm (equivalent to 0.83 CD) while the annular jet penetrates the armored steel target with a thickness of 60.0 mm (equivalent to 0.53 CD). The numerical simulation is in agreement with the experimental data. The research results are of great significance in guiding the design of the front-stage annular shaped charge structure of the multi-effect destructive warhead.

## Figures and Tables

**Table 1:** Parameters of the explosive material model and the corresponding JWL state equation [16].

ρ/(g·cm^−3^)	A/GPa	B/GPa	R_1_	R_2_	ω	E/(J·mm^−3^)
1.713	524.2	7.768	4.2	1.1	0.34	8.499

**Table 2:** Parameters of the red copper and armored steel models [16,17].

Material	ρ/(g·cm^−3^)	A	B	C	n	m
**Red copper**	8.96	0.09	0.29	0.02	0.31	1.09
**steel**	7.896	350.0	275.0	0.022	0.36	1.0
**Armored steel**	7.83	1450	382.5	0.018	0.245	1.06

**Table 3:** The table of values of various factor levels.

Level	Factor		
	L/CD	α (°)	β (°)
1	0.5	20	80
2	1.0	40	90
3	1.5	60	100
4	2.0	80	110

**Table 4:** Calculation results of the orthogonal optimization simulation.

No.	L/CD	α (°)	β (°)	P (mm)	D_h_ (mm)
1	0.5	20	80	31.5	99.4
2	0.5	40	90	22.0	99.4
3	0.5	60	100	18.5	110.6
4	0.5	80	110	12.9	149.2
5	1.0	20	90	20.5	120.0
6	1.0	40	80	25.5	91.6
7	1.0	60	110	34.2	126.4
8	1.0	80	100	21.0	137
9	1.5	20	100	19.6	133.8
10	1.5	40	110	20.7	136.6
11	1.5	60	80	62.0	91.6
12	1.5	80	90	20.7	115.4
13	2.0	20	110	17.8	140.8
14	2.0	40	100	25.8	128.4
15	2.0	60	90	62.1	96.6
16	2.0	80	80	21.0	115.6

**Table 5:** Results of data processing and calculation.

	P (mm)			D_h_ (mm)		
	L/CD	α (°)	β (°)	L/CD	α (°)	β (°)
K1	81.9	86.7	150.7	458.6	504	398.2
K2	101.2	126.5	125.6	475	466	445.4
K3	120.4	176.5	84.9	476.4	425.2	519.8
K4	126.4	74.3	85.2	481.4	531.2	563
K1/4	20.5	21.7	37.7	114.7	126	99.6
K2/4	25.3	31.6	31.4	118.8	116.5	111.4
K3/4	30.1	44.1	21.6	119.1	106.3	130.0
K4/4	31.6	18.6	21.3	120.0	132.8	140.8
S	11.1	25.5	16.6	5.3	26.5	41.2

**Table 6:** The comparison of the numerical simulation and experiment results.

Result	Opening Diameter	Existing Diameter	Penetration Depth
Numerical simulation	94.3 mm	81.6 mm	60.0 mm
Experiment	95.0 mm	86.3 mm	60.0 mm

## Data Availability

The data that support the findings of this study are available from the corresponding author (J.F.), upon reasonable request.
